# Multi-Strain Infections and ‘Relapse’ of *Leucocytozoon sabrazesi* Gametocytes in Domestic Chickens in Southern China

**DOI:** 10.1371/journal.pone.0094877

**Published:** 2014-04-11

**Authors:** Wenting Zhao, Baowei Cai, Yanwei Qi, Shengfa Liu, Lingxian Hong, Mingke Lu, Xin Chen, Chunhui Qiu, Wenfeng Peng, Jian Li, Xin-zhuan Su

**Affiliations:** 1 State Key Laboratory of Cellular Stress Biology, School of Life Sciences, Xiamen University, Xiamen, Fujian, the People's Republic of China; 2 Laboratory of Malaria and Vector Research, National Institute of Allergy and Infectious Diseases, National Institutes of Health (NIH), Bethesda, Maryland, United States of America; 3 College of Life Sciences, Fujian Agriculture and Forestry University, Fuzhou, Fujian, the People's Republic of China; Institut Pasteur, France

## Abstract

*Leucocytozoon* parasites infect many species of avian hosts, including domestic chicken, and can inflict heavy economic loss to the poultry industry. Although the prevalence and distribution of two *Leucocytozoon* species (*L. sabrazesi* and *L. caulleryi*) have been reported in China previously, there are many questions related to the parasite infection that remain unanswered, including population diversity and transmission dynamics in domestic chickens. Here we surveyed chicken blood samples from seven sites in four provinces of China to identify *Leucocytozoon* infection, characterized parasite diversity within individual infected hosts and between sampling sites, and investigated the dynamics of gametocytemia in chickens over time. We found high infection rates in three of the seven sites. Clustering parasite sequences of the mitochondrial cytochrome oxidase III (*coxIII*) and cytochrome b (*cytb*) genes showed lack of grouping according to geographic origins and individual hosts carrying large numbers of *L. sabrazesi* strains. Monitoring gametocytemia in blood samples from infected chickens over time showed ‘relapse’ or persistence of low-level gametocytemia for 4–5 months, which could be explored as an *in vivo* model for testing drugs against liver stages of Apicomplexan parasites. This study provides important information on population diversity and transmission dynamics of *L. sabrazesi* and for disease control.

## Introduction

*Leucocytozoon* is a genus of parasitic protozoa belonging to the phylum Apicomplexa that infects numerous species of avian hosts, including the domestic chickens, pigeons, owls, and penguins [1]–[7]. The parasites have a complex life cycle, generally characterized by having merogony in fixed tissues (such as liver) of vertebrate hosts, sexual differentiation (gametocytes) in blood cells, and sporogony in simuliid flies or culicoiddes midges [1]. Reported symptoms of birds infected with *Leucocytozoon spp* include listlessness, green feces, anorexia, anemia and even death [1], [8]–[10]. Pathological changes such as edema caused by the presence of large schizonts of *L. caulleryi* in reproductive organs can lead to decreased egg production [11]. Pink pigeons infected with *L. marchouxi* were significantly less likely to survive to 90 days post-sampling [4]. Mortality rates of 35%–56% have also been reported among domestic chickens raised in cages [12].

Although both *L. sabrazesi* and *L. caulleryi* have been reported in Fujian and other provinces of China, the majority of the previous studies, however, were surveys of *Leucocytozoon* infection and species identification based on blood smear detection of the circulating gametocytes [12]–[15]. These studies have provided important information for the understanding of the disease prevalence and for disease control, but many critical questions on parasite transmission dynamics remained to be answered. For example, it is important to investigate whether a chicken host is infected with a single parasite strain or with mixed populations of different parasite strains or species, and whether infection with multiple parasite strains and/or species has any effect on pathogenesis. Birds mixed-infected with different *Leucocytozoon* species or *haemosporidian* lineages and human hosts infected with different species or strains of *Plasmodium* parasites have been reported [16]–[20]. Another important question is how long an infected chicken host can carry gametocytes in the blood or act as a reservoir to transmit the parasite? Many *Plasmodium* parasites such as the human malaria parasite *P. vivax* and bird malaria parasite *P. relictum* are known to have relapses that can produce rounds of blood infections over a long period of time [21], [22]. Do the *Leucocytozoon* parasites release gametocytes into the blood stream continuously or intermittently? These issues are important for a better understanding of the transmission dynamics of the parasite and for providing guidance for disease control.

In this study, we first conducted field surveys of *Leucocytozoon* infection in domestic chickens at seven locations in China using blood smear and microscopic observation. Based on DNA sequences from the parasite mitochondrial cytochrome oxidase III (*coxIII*) and cytochrome b (*cytb*) genes, we showed that an individual chicken host was generally infected with multiple parasite strains regardless the origin of the host. Additionally, no obvious clustering of parasites according to their geographic origins was observed, suggesting high transmission and/or high mutation rates. Monitoring gametocytemia from blood samples in infected chickens showed potential ‘relapse’ and presence of gametocytes over a long period of time, which could be explored as animal models for screening anti-liver stage or anti-sexual stage drugs. These results suggest high transmission rates with multiple infective bites during the chicken host's lifetime and infection of more than one genotype (strains) in a single infective bite.

## Materials and Methods

### Sampling sites and blood collection

**Figure 1:**
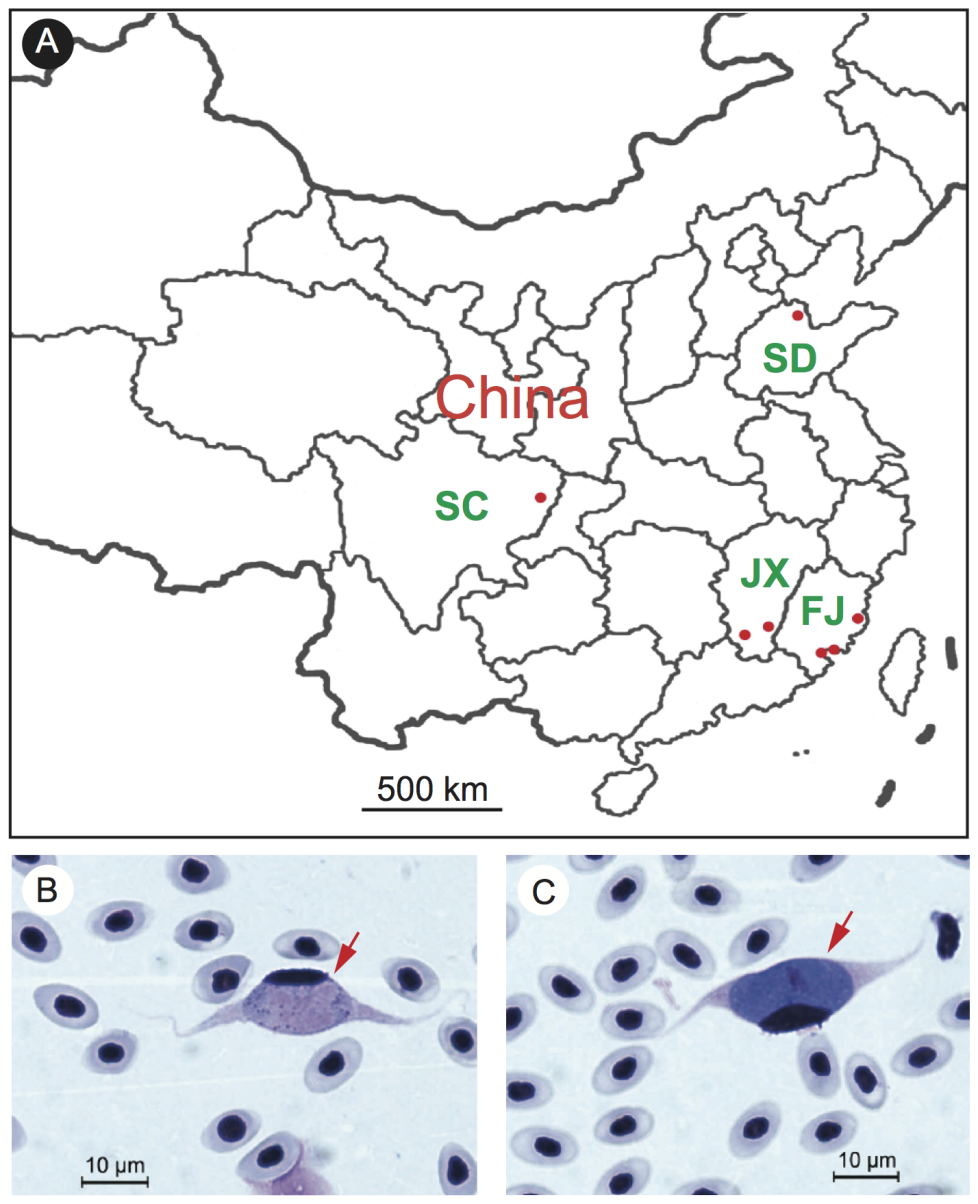
Sampling locations and images of host blood cells infected with *Leucocytozoon* gametocytes. A, a partial map of China showing sampling locations (red dots) of this study; B, an image of blood smear showing a male gametocyte; C, an image of blood smear showing a female gametocyte. Red arrows point to blood cells infected with gametocytes.

Chicken blood samples were collected from three sites in Fujian province, two sites in Jiangxi province, one site in Sichuan province, and one site in Shandong province of China (**Figure 1A**). The chickens were raised in indoor cages, free-range farms, or in the backyards of individual households. Two to three hundreds µl of blood were collected from the wing veins and were immediately mixed with 40 µl anticoagulant solution (8 g/L citric acid and 22 g/L trisodium citrate, pH 7.2) in 1.5 ml centrifuge tube. Thin blood smears were made, air-dried, fixed with 100% methanol, and then stained with Giemsa stain. The gametocytemia was estimated as the proportion of infected cells after counting 10,000 red blood cells (RBCs). The experiments were conducted under a protocol (#XMULAC20130065) approved by the Animal Care and Ethics Committee at Xiamen University. For monitoring gametocytemia over time, three positive chickens were kept in a room with screened windows, and thin blood smears were made at 3–7 days intervals for approximately five months. Additionally, no simuliid vectors were found near the university campus.

### DNA extraction and amplification

Blood cells were lysed using saponin solution before DNA extraction. Briefly, 10 µl blood were suspended in 600 µl 0.1% saponin in PBS (pH 7.4) and kept on ice for 5 min; the parasites and nuclei of host cells were pelleted by centrifugation at 5000 rpm for 3 min and then washed with PBS (3X). The pellet were resuspended in 400 µl lysis buffer (50 mM TrisCl, 100 mM EDTA, 2% SDS; pH 8.0) and incubated overnight at 50°C. DNA was extracted using phenol-chloroform method and dissolved in ddH_2_O.

After DNA extraction, a series of PCRs were performed using primers synthesized based on published sequences of the *L. sabrazesi* mitochondrial DNA (Accession No. AB299369.1). The primers used to amplify an 869 bp DNA segment of the *coxIII* gene (LscoxIII-F: 5′GAACACAATAATGGGTTGTCA3′and LscoxIII-R: 5′GAACCTTCTTACCGTTATATG′3) and a 666 bp *cytb* sequence (Lscytb-F1:5′TAATCACATGGGTTTGTGGA3′; Lscytb-R: 5′GAGAGCTGTAATCATAGTGT3′) from the parasite were purchased from Sangon Biotech Co., Ltd. (Shanghai). All PCR amplifications were performed in a 20 µl volume containing 4 µl of genomic DNA (∼50 pg), 80 µM dNTPs, 0.8 mM MgCl_2_, 0.1 µM of each primer, 1 unit of Taq DNA polymerase and were run using the following cycling conditions: initial denaturation at 94°C for 2 min; then cycling 20 s at 94°C, 20 s at 50°C, 30 s–90 s (depending on product sizes) at 68°C, for 40 cycles; and a final step at 68°C for 2 min. PCR products (4 µl each) were separated on 1–2% agarose gels, visualized on a UV light box and photographed using a Tanon-2500(R) Gel Imaging System (Tanon).

### DNA cloning and sequencing

The PCR products were purified from agarose gels and sent for commercial sequencing (Invitrogen, China). Some PCR products were also cloned into the Pmd18-T vector for sequencing according to the manufacture's protocol (Takara). Briefly, ligation products were used to transform TOP 10 high efficiency competent cells and were plated onto LB/ampicillin/IPTG/X-Gal plates. Multiple positive (white) colonies were picked randomly from each cloned sample. DNAs from the colonies were sequenced after restriction digestion verifying the presence of inserts. The DNA sequences were initially aligned and edited using BioEdit [23].

### Sequence clustering analysis

To investigate the relationship between the obtained and published sequence of the two genes, we aligned the sequences using Clustal W and clustered the *cytb* and *coxIII* sequences using the neighbor-joining method implemented in the program MEGA5 [24]. Pairwise comparison of the numbers of base substitutions per site and the standard errors from between the *coxIII* sequences were also estimated using the same software. Bootstrap values were estimated after 1,000 replications.

## Results

### Prevalence of *Leucocytozoon spp* in domestic chicken in China

To investigate the current prevalence of *Leucocytozoon* infection in China, we smeared 212 chicken blood samples from seven locations including: Binzhou of Shandong province; Dazhou of Sichuan province; Ruijin and Ganzhou of Jiangxi province; Fuzhou, Zhangzhou and Haicang of Fujian province (**Figure 1A** and **Table 1**). Infected blood smears showed elongated, spindle-shaped blood cells containing gametocytes with host nuclei at one side of the cells characteristic of infections with many *Leucocytozoon spp* (**Figure 1B and 1C**). Fifty-nine of the 212 chickens (27.8%) from four of the seven sampling sites were positive for *Leucocytozoon* gametocytes after microscopic examination, with infection rates ranging from 2.1% to 100% among the positive sites (**Table 1** **and Table S1**). The percentage of infected blood cells (gametocytemia) also varied widely, ranging from 0.01‰ to 2.87‰. The results showed high infection rates and high prevalence in domestic chickens in southern China. Based on the morphology of the elongated host blood cells carrying gametocytes, the parasites appeared to be *L. sabrazesi* as described previously [14].

**Table 1:** Survey of *Leucocytozoon* infections in domestic chickens in China.

Location	Type of farm	Time of sampling	No. birds Exam.	No. birds infected	Positive rate (%)	Ave. ‰ gam. (±SD)
Zhangzhou 1, Fujian	Free-range	11-Dec-12	25	17	68	0.10 (0.13)
Zhangzhou 2, Fujian	Free-range	25-Aug-13	10	7	70	0.59 (1.03)
Haicang 1, Fujian	Free-range	23-Mar-13	13	9	69.2	0.69 (0.33)
Haicang 2, Fujian	Free-range	1-Apr-13	3	3*	100	0.39 (0.54)
Haicang 3, Fujian	Free-range	5-Jul-13	7	7	100	0.86 (1.10)
Haicang 4, Fujian	Free-range	26-Oct-13	10	9	90	0.34 (0.34)
Fuzhou 1, Fujian	Backyard	29-Mar-13	11	0	0	—
Fuzhou 2, Fujian	Indoor farm	11-Oct-13	97	2	2.1	—
Ganzhou, Jiangxi	Backyard	9-Feb-13	8	5	62.5	0.23 (0.18)
Ruijin, Jiangxi	Backyard	10-Mar-13	10	0	0	—
Dazhou, Sichuan	Backyard	8-Feb-13	10	0	0	—
Binzhou, Shandong	Backyard	16-Feb-13	8	0	0	—
Sum			212	59		

Location, names of sampling locations, district/city and province; No. birds Exam, numbers of chicken examined;

No. birds infected, numbers of chicken infected; positive rate, percentage of chicken infected with

*Leucocytozoon*; Ave. ‰ gam. rate (±SD), averaged numbers of gametocytes per 1000 red blood cells; SD,

standard deviation. * Initial smear from one chicken was negative, but gametocytes were found in subsequent examinations.

### Mixed infections of diverse genotypes in an individual host

**Figure 2:**
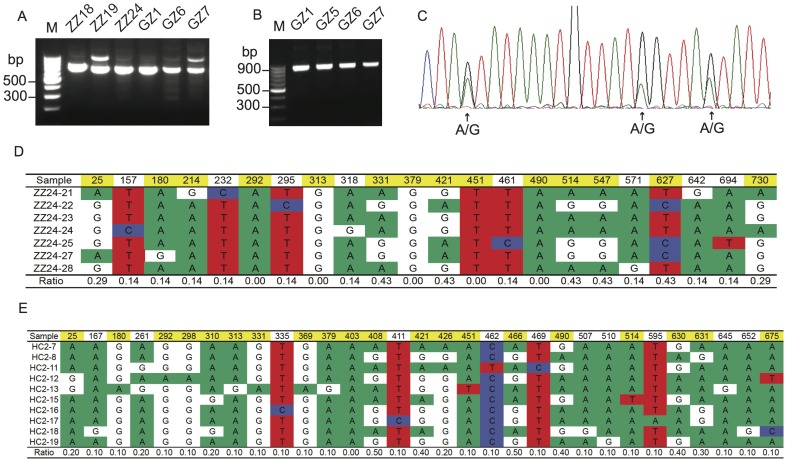
Agarose gels showing PCR amplified products from individual chicken hosts and mixtures of DNA sequences from single chicken hosts. A, Amplified *cytb* segments from six chickens obtained from two locations: ZZ, Zhangzhou of Fujian province; GZ, Ganzhou of Jiangxi province; B, PCR products of *coxIII* from four chickens from Ganzhou of Jiangxi province; C, an electropherogram of a small segment of the *coxIII* from PCR product direct sequencing showing three positions with mixed signals (arrows, A and G); D, polymorphic positions in the *coxIII* gene segment amplified and sequenced from chicken ZZ24. The positions highlighted in yellow represent positions having two peaks (alleles) in the sequence from PCR product direct sequencing. Samples ZZ24-21 to ZZ24-28 are individual sequences after the PCR products were cloned into TA-vector and sequenced; E, another example as in D from chicken HC2. Ratio, the ratios of minor alleles at each position.

To confirm the identity of the parasite, we extracted DNA from the infected chicken blood samples and amplified a 666 bp fragment of the *cytb* gene and an 869 bp segment from the *coxIII* gene of the parasite mitochondrial genome from parasites collected from Zhangzhou and Haicang of Fujian province and Ganzhou of Jiangxi province using *L. sabrazesi* specific primers as described in the **Methods**. For most samples, a band of expected size was amplified using *L. sabrazesi*-specific primers (**Figure 2A and 2B**). The PCR products of *cytb* and *coxIII* from selected chicken blood samples were initially sequenced directly without cloning into a plasmid vector. Although the DNA sequencing results clearly showed that both *cytb* or *coxIII* gene sequences belonged to those of *Leucocytozoon* after comparison with known sequences in public databases, many positions in the sequences had two overlapping electropherogram peaks, suggesting the presence of more than one alleles or infections with multiple strains (**Figure 2C**). For some of the samples, a secondary PCR product larger than the expected size was also present in the *cytb* amplification products (**Figure 2A**). However, sequencing of the larger *cytb* product did not identify any known sequences in the public databases, and the origin of the DNA was not investigated further.

**Figure 3:**
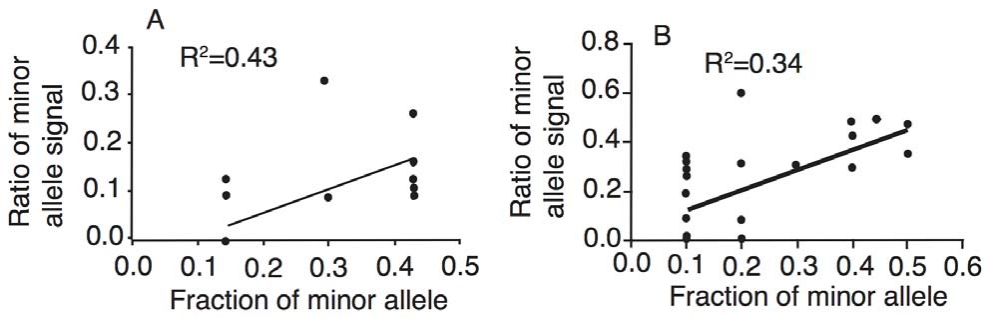
Correlation plot of ratios of minor alleles of *coxIII* gene segment from two chicken hosts. A, Plot of allelic ratios from chicken ZZ24. Minor allele ratios from seven individual sequences were plotted against electropherogram signal ratios of PCR product direct sequencing; B, the same as A, but from 10 individual sequences of chicken HC2. The X-axis is the ratios of minor alleles from individual clones, and the Y-axis is the ratios of peak signals [minor peak/(minor+major peak)] from the sequence of direct PCR product sequencing. Positions without two alleles in the bacterial colony sequences are not included.

The mixed peaks in the electropherograms from direct PCR product sequencing suggested infections with multiple parasite strains in a single chicken. To obtain individual sequences, we cloned the *cytb* PCR products from nine infected chickens and the *coxIII* PCR products from eight chickens into the Pmd18-T cloning vector. We obtained DNA sequences from 74 bacterial colonies and found that 58 of the 74 sequences were unique (78.4%), suggesting from different parasite variants (**Table 2**). Alignment of the sequences indeed showed substitutions at various positions of individual cloned sequences (**Figure S1 and Figure S2**). For example, alignment of seven *coxIII* sequences (only 747 bp coding region used) from chicken ZZ24 of Zhangzhou, Fujian province, showed 22 polymorphic sites (2.9%) between the individual cloned sequences and the sequence obtained from PCR product direct sequencing (**Figure 2D** **and Figure S1**). Among the 22 polymorphic sites, 14 positions (63.6%) were detected both in the sequence from PCR product direct sequencing and one or more individual cloned sequences, suggesting that these sites were not from PCR artifacts (**Figure 2D**). Sequencing a PCR product directly generally will not see PCR-generated mutations because even a random mutation is introduced into a PCR product by Taq polymerase in the first round of amplification, the ratio of the random mutation to the initial template population remains small and will not be detected in the sequencing. There were five positions (292, 313, 379, 451, and 490) having two alleles in the sequence of PCR product direct sequencing, but no alternative alleles were found in the individual cloned sequences. Sequencing additional clones will likely identify the missing clones. On the other hand, there were also eight cloned sequences that had a second allele not present in the sequence of PCR product direct sequencing. Similarly, 35 polymorphic sites (4.7%) were found in the same *coxIII* sequences from chicken HC2 of Haicang, Fujian province (**Figure 2E**). Among the polymorphic sites, 21 sites (60.0%) had two alleles that were matched between the sequence of PCR product direct sequencing and the 10 unique individual cloned sequences. Fourteen sites had two alleles in one or more individual sequences, but only one allele was found in the sequence from PCR product direct sequencing. In contrast, only position 403 had two alleles in the sequence of PCR product direct sequencing, and no alternative allele was found in the individual sequences. Compared with those from chicken ZZ24, the reduction in the number of positions with mixed alleles in the HC2 sequence of PCR product direct sequencing (one from HC2 compared with five from ZZ24) and the increase in the number of polymorphic sites in the individual sequences (14 in the HC2 *vs* 8 in the ZZ24) were likely due to the fact that more individual colonies from HC2 (10) than those from ZZ24 (7) were sequenced. Additionally, plot of minor allele ratios from individual cloned sequences against the signal ratios of minor allele electropherogram peaks from direct PCR product sequencing showed some degree of positive correlation (**Figure 3**). These results supported a conclusion that the majority of the substitutions detected (>60%, at least those matching polymorphic sites from PCR product direct sequencing) were from mixed infections of different parasite strains, not from PCR introduced artifacts.

**Table 2:** Summary of blood samples sequenced and the number of unique mitochondrial cytochrome oxidase III (*coxIII*) and cytochrome b (*cytb*) sequences obtained.

Sample	Sampling site	Sampling date	Amplified gene	No. clones obtained	No. independent clones
ZZ18	Zhangzhou	11-Dec-12	*CoxIII*	2	2
ZZ19	Zhangzhou	11-Dec-12	*CoxIII*	7	7
ZZ24	Zhangzhou	11-Dec-12	*CoxIII*	7	7
GZ1	Ganzhou	26-Oct-13	*CoxIII*	2	1
GZ6	Ganzhou	26-Oct-13	*CoxIII*	3	2
GZ7	Ganzhou	26-Oct-13	*CoxIII*	4	3
HC2	Haicang	9-Feb-13	*CoxIII*	10	10
HC4	Haicang	9-Feb-13	*CoxIII*	1	1
ZZ18	Zhangzhou	11-Dec-12	*Cytb*	5	1
ZZ19	Zhangzhou	11-Dec-12	*Cytb*	3	2
ZZ24	Zhangzhou	11-Dec-12	*Cytb*	4	2
GZ1	Ganzhou	26-Oct-13	*Cytb*	5	4
GZ6	Ganzhou	26-Oct-13	*Cytb*	3	2
GZ7	Ganzhou	26-Oct-13	*Cytb*	3	3
HC2	Haicang	9-Feb-13	*Cytb*	5	3
HC4	Haicang	9-Feb-13	*Cytb*	5	4
HC8	Haicang	9-Feb-13	*Cytb*	5	4
Total				74	58

Note: ZZ, Zhangzhou; HC, Haicang; GZ, Ganzhou.

### Clustering *cytb* and *coxIII* sequences and lack of geographic differentiation

**Figure 4:**
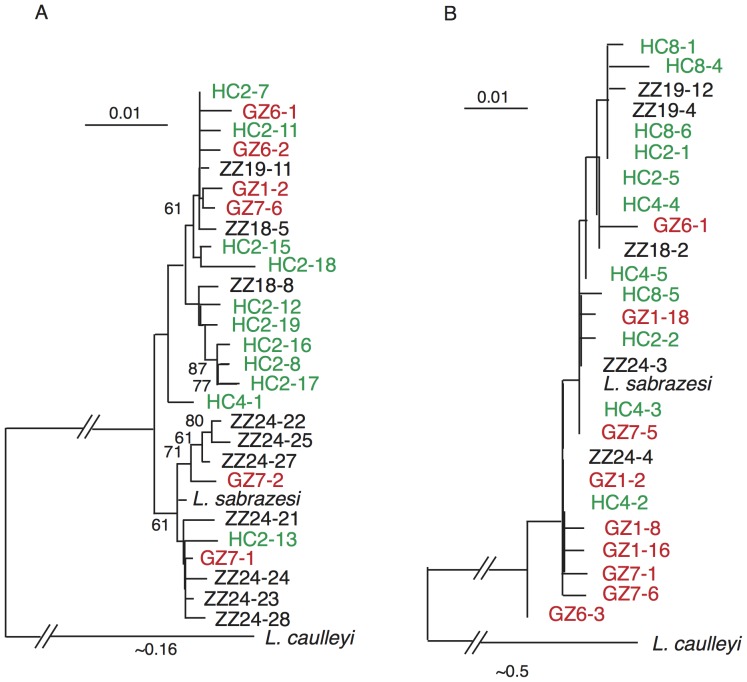
Dendrograms of the *coxIII* and *cytb* gene segments clustering parasite strains cloned from chickens obtained from three locations in southern China. A, clustering of the *coxIII* sequences; B, clustering of the *cytb* sequences. HC and ZZ are Haicang and Zhangzhou of Fujian Province; GZ is Ganzhou of Jiangxi province. Both *L. sabrasesi* and *L. caulleryi* sequences were downloaded from NCBI database with accession number AB299369.1 and AB302215.1, respectively. The dendrograms were produced using the neighbor-joining method implemented in the program MEGA5. Bootstrap values higher than 60% after 1,000 permutations are as labeled.

Hierarchical clustering of the amplified individual *coxIII* and *cytb* sequences showed grouping of the sequences with the *L. sabrazesi coxIII* and *cytb* sequences (accession #AB299369.1) in public databases, not with those of *L. caulleryi* (**Figure 4A and 4B**), confirming that the parasites were all *L. sabrazesi*, consistent with the classification based on morphology. Although the topology of the ‘trees’ of the *coxIII* and *cytb* genes were different, with longer branches produced from the *coxIII* sequences, the distribution of individual clones derived from a single host (or one location) were similar: the DNA sequences from one chicken were placed in different clusters or mixed-clustered with sequences from different geographic regions. For example, sequences from HC2 were placed in different clusters, with HC2-13 clustered with sequences from ZZ24 and GZ7, HC2-7/11 clustered with GZ1/6/7 and ZZ18/19, and HC2-8/12/16/17/19 clustered by themselves in the *coxIII* tree (**Figure 4A**). Similar patterns were seen in the *cytb* tree even though the sequences appeared to be less polymorphic, and the short branches did not have significant bootstrap values (**Figure 4B**). Furthermore, estimates of pairwise genetic distance from the *coxIII* gene also showed large genetic distances between the parasite clones from infected chickens of the same sampling sites, or even from the same host (**Table S2**). For example, the genetic distance between clones GZ7-2 and GZ7-6 (two clones from chicken GZ7) was 0.0221 that is larger than the largest averaged genetic distances (0.0188) between three sampling sites (Zhangzhou, Haicang, and Ganzhou) (**Table S3**). These results suggest a lack of geographic differentiation for samples collected from several hundred kilometers apart, which could be due to high rates of mutation and population admixture of parasites carried by migrating birds.

### The dynamics of gametocytemia over time (‘relapse’ pattern)

**Figure 5:**
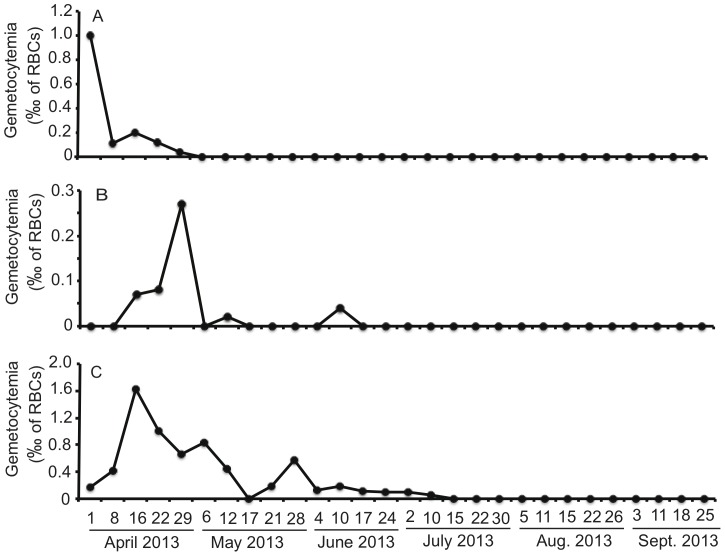
Gametocytemia in infected chicken hosts over time. Three infected chickens were monitored for the presence of gametocytes, expressed as numbers of gametocytes per 1,000 red blood cells (RBCs), in Giemsa stained blood smears at 4–7 days intervals for 5 months. A, Gametocytemia from chicken HC1; B, gametocytemia from chicken HC2; C, gametocytemia from chicken HC3. Blood sampling dates are as marked.

The duration of the presence of gametocytes in the blood stream is an important factor affecting the transmission and prevalence of the disease. To investigate the dynamics of circulating gametocytes over time, we monitored the gametocytemia in three infected chickens from Haicang (HC1, HC2, and HC3, sampled on April 1, 2013) at 4–7 days intervals for ∼5 months. The initial gametocytemia from the three chickens were 1.0‰, 0‰, and 0.17‰, respectively (**Table S1 and** **Figure 5**). Interestingly, the gametocytemia of HC2 and HC3 increased to peak at 0.27‰ on week 5 and 1.63‰ on week 3, respectively, before declined to <0.01‰. Two additional gametocytemia peaks were observed on week 7 and 12 in HC2, and a major peak of 0.57‰ gamecytoemia was found on week 10 in HC3. The gametocytemia were low or undetectable for both chickens before another small rebound of ∼0.01‰ gametocytemia on August 5 (around week 20) (**Table S4**). For HC1, the gametocytemia declined from 1.0‰ to 0.04‰ on April 29 (week 5), then declined to 0% round on June 4 (week 10). These results suggested that the infected hosts can act as reservoirs for transmission at least for 4 months and showed that the release of gametocytes was not constant, representing a pattern similar to the ‘relapse’ observed in the human malaria parasite *P. vivax* or bird malaria parasite *P. relictum*.

## Discussion

This study examined *Leucocytozoon* infection in domestic chickens from seven sites in four provinces of China. Three sites in Fujian province and one site in Jiangxi province were positive for *L. sabrazesi*. The lack of infected chickens at the other sites could be due to the relatively small numbers of birds surveyed. Another possibility was the timing of the blood collection. There might be no active transmission at the time of our blood collection at the sites in the northern provinces during February and March because of relatively low temperatures and the absence of insect vectors (The temperatures could be near or under 0°C in Sichuan and Shandong provinces). Nonetheless, quite high infection rates were found at the three of the four sites with positive blood samples, consistent with those reported in previous surveys [12]–[15]. Further studies surveying larger numbers of chickens during the months of higher temperatures in the spring and summer may be necessary to determine the prevalence and the transmission dynamics at the negative sampling sites.

One of the most interesting observations of this study was the infection of parasites with diverse genetic backgrounds in a single host. For example, most of the seven and 10 colonies randomly picked from cloned PCR products from chicken ZZ24 and HC2, respectively, contained unique *coxIII* gene sequences (**Figure 2** **and Table S2**), suggesting highly diverse populations of parasite strains in the hosts. The numbers of parasite strains could be higher if more colonies were sequenced because there were additional positions of mixed alleles in the sequence from PCR product direct sequencing not represented in the individual cloned sequences. Amazingly, all the samples sequenced, three each from each of three sites (Haicang and Zhangzhou of Fujian province; Ganzhou of Jiangxi province), were mixed-infected with multiple parasite strains (**Figure 4** **and** **Table 2**). The mixed genotypes in each bird suggested that the host had received multiple infective bites during a transmission season and/or a single infective bite containing more than one parasite strain at the same time. These results suggest high transmission rates with multiple infective bites per year and/or infective bites of more than one strain in the sampling area. Mixed-lineage infections of different species of *Plasmodium, Leucocytozoon, and Haemoproteus* parasites that showed no geographic specificity and had broad geographic distributions have been reported [19], [25]; however, our study represent the first report of mixed strains (intra-species) infection of *L. sabrazesi* in domestic chicken in southern China. It would be interesting to investigate the effects or consequences of multi-strain or multi-species infections on the manifestation of disease symptoms.

There were positions that had two alleles in individual cloned sequences but the alternative minor alleles were not found in the sequences of PCR product direct sequencing. There are two potential explanations for the observations: One possibility is that the minor allele clones came from low frequency strains, but the signals of the minor alleles were too weak to be detected in the uncloned PCR products by the Sanger's sequencing. This was likely the case because 19 of the 20 cases had only one clone (out of seven or 10) carrying the alternative allele, suggesting that the minor alleles had low frequencies. A minor allele of <10% in a DNA mixture will unlikely be detected by the Sanger sequencing method or microsatellite typing [26]. Another possibility is that the polymorphic sites were simply PCR artifacts. Taq polymerase is known to introduce errors during PCR amplification. There are two questions associated with potential PCR artifacts. First, how many of the observed polymorphic sites were due to PCR errors? Second, how much will the PCR errors affect study conclusions? To answer the first question, we can estimate the numbers of potential errors using the known enzyme error rate from the enzyme provider (Thermo Scientific). The enzyme we used has an error rate of 2.2×10^−5^ errors per nucleotide per cycle. We amplified 40 cycles, giving an error rate of 8.8×10^−4^ per nucleotide per amplification; or in theory we'll see an error per 1136.4 bp. The *coxIII* fragment is 747 bp, and on average, we should see 0.65 bp errors per copy. According to this error rate, we would expect to have ∼11 errors [(747×17)/1136.4 = 11.2] among the 57 polymorphic sites detected from the 17 sequences (7 from ZZ24 and 10 from HC2), suggesting ∼80% (46/57) of true polymorphic sites. As discussed above, the sites of mixed alleles in the PCR product direct sequencing are considered true polymorphisms because errors introduced by Taq polymerase are randomly distributed in each individual product, and the proportion of the random mutation to the initial template population is small. Consequently, the major sequencing signal at any one nucleotide will be from the original DNA templates with no mutations. Among the 57 sites detected from chicken ZZ24 and HC2, 35 (61.4%) had mixed alleles in PCR product direct sequencing, which was within the range of the estimated rate of true differences (∼80%) using the enzyme error rate. To answer the second question, we removed the polymorphic sites in the individual cloned *coxIII* sequences not matched with the 35 sites in the sequences of PCR product direct sequencing from ZZ24 and HC2, creating individual sequences with polymorphic sites verified by PCR product direct sequencing. We then performed hierarchical clustering and estimated the genetic distances between the cloned sequences. We obtained a dendrogram similar to that in Figure 4A (**Figure S3**), *i.e.* clustering HC2-13 more closely with parasites from ZZ24 than those of HC2 and sequences with large genetic distances between clones from the same host (**Table S5**). Therefore, the conclusions of infections with highly diverse parasite strains in chickens of the same sampling sites or even in a single host would not be changed even if the potential errors by Taq polymerase were removed.

In addition to mixed infection, the period of circulating gametocytes in the blood stream of an infected host is another factor that can contribute to high prevalence and mixed-infections. Monitoring gametocytemia in blood smears showed continuous presence of low-level gametocytes in the infected host over several months with fluctuations of gametocytemia (‘relapse’). Generally, when infected chickens were first identified, the gametocytemia were relatively high, but the gametocytemia then dropped to low (under 0.01‰) or undetectable levels in the blood smears after 2–3 months. The observation of high gametocytemia at the time of first smear followed by low-levels of gametocytemia in the chickens suggests that the high gametocytemia could be due to active and constant infections of the chicken hosts. Another possibility for the observed high gametocytemia was that the parasites represented new infections before the development of immunity that later could suppress the parasitemia. The re-appearance (or increase in gametocytemia) of gametocytes in chicken HC2 and HC3 resembled the ‘relapse’ observed in other parasites such as the malaria parasite *P. relictum*. This presence of gametocytes in the blood stream for several months certainly can provides a parasite source for continuous transmission and contributes to the high transmission rates and mixed infections.

Microscopic examination of blood smears is a simple and reliable method for detecting parasite infection. However, the sensitivity of microscopy is depended on the number of cells counted each slide and the experience of the person who reads the slide, and our data could under-estimate the period of circulating gametocytes. Additionally, monitoring chicken hosts over a period of time may not detect infections with low gametocytemia at the time of examination, as exemplified by HC2 of the April 1, 2013 survey (**Figure 5** **and** **Table S4**). Development of more sensitive methods such as PCR-based amplification to detect parasite DNA in circulation may provide more accurate estimates of transmission potential during the life of an infected host. Nonetheless, our results showed high prevalence of *L. sabrazesi* infection in domestic chickens in southern China and the presence of a large number of parasite strains in an individual infected host. The possibility of infection with multiple infective bites with more than one parasite strain as well as long distance migration of wild birds could play an important role in parasite population diversity and structure, which could present difficulties in disease control and containment of drug resistance.

In additional to being an important pathogen, *Leucocytozoon* can be a valuable model for studying host-parasite interaction. For example, the parasites can transform the host blood cells into spindle-shaped cells. It would be interesting to investigate whether the infected cells can still function properly during infection. Elucidating the mechanism and signaling pathways during host cell transformation by the parasite may provide important information for disease control and for understanding host cell differentiation and development. The availability of large numbers of parasite strains with unique genotypes will provide plentiful resources of parasite clones for genetic crosses and for genetic mapping genes controlling important parasite traits. Finally, the presence of merozoites in the liver for a long period of time and the ‘relapse’ of gametocytemia could be explored as an *in vivo* system for testing drugs against tissue stages or gametocytes of Apicomplexan parasites.

## Supporting Information

Figure S1 **DNA sequence alignments of the** ***Leucocytozoon sabrazesi coxIII*** **gene from chickens obtained from three locations in southern China.**
(TIFF)

Figure S2 **DNA sequence alignments of the** ***Leucocytozoon sabrazesi cytb*** **gene from chickens obtained from three locations in southern China.**
(TIFF)

Figure S3 **Dendrograms of the** ***coxIII*** **gene segment clustering parasite strains cloned from chickens ZZ24 and HC2.** The clustering was performed as in Figure 4 except using the 35 polymorphic sites verified by direct PCR product sequencing.
(TIFF)

Table S1 **Surveys of** ***Leucocytooon*** **infections in domestic chickens from seven locations in China.**
(XLSX)

Table S2 **Estimates of pairwise genetic distance of the** ***Leucocytozoon sabrazesi coxIII*** **gene.** The numbers of base substitutions per site (bottom half) and standard errors (top half) were estimated using Tamura-Nei model implemented in MEGA5. The coloring was done using Excel conditional formatting for easy viewing. HC and ZZ are chickens from Haicang and Zhangzhou of Fujian Province, respectively; GZ are chickens from Ganzhou of Jiangxi province.
(XLSX)

Table S3 **Estimates of average genetic distances of the** ***Leucocytozoon sabrazesi coxIII*** **gene between three sampling sites.**
(XLSX)

Table S4 **Gametocytemia in three infected chickens over time.**
(XLSX)

Table S5 **Estimates of pairwise genetic distance of the** ***Leucocytozoon sabrazesi coxIII*** **gene (747 coding segment) using 17 cloned sequences from chicken ZZ24 and HC2 after removing potential errors introduced by Taq polymerase.** The analyses were performed as those in Table S2.
(XLS)
