# Supplementary figures and images for: Multi-Strain Infections and ‘Relapse’ of Leucocytozoon sabrazesi Gametocytes in Domestic Chickens in Southern China
Source: PLoS One. 2014 Apr 11;9(4):e94877. doi: 10.1371/journal.pone.0094877 (PMC3984278; doi:10.1371/journal.pone.0094877)

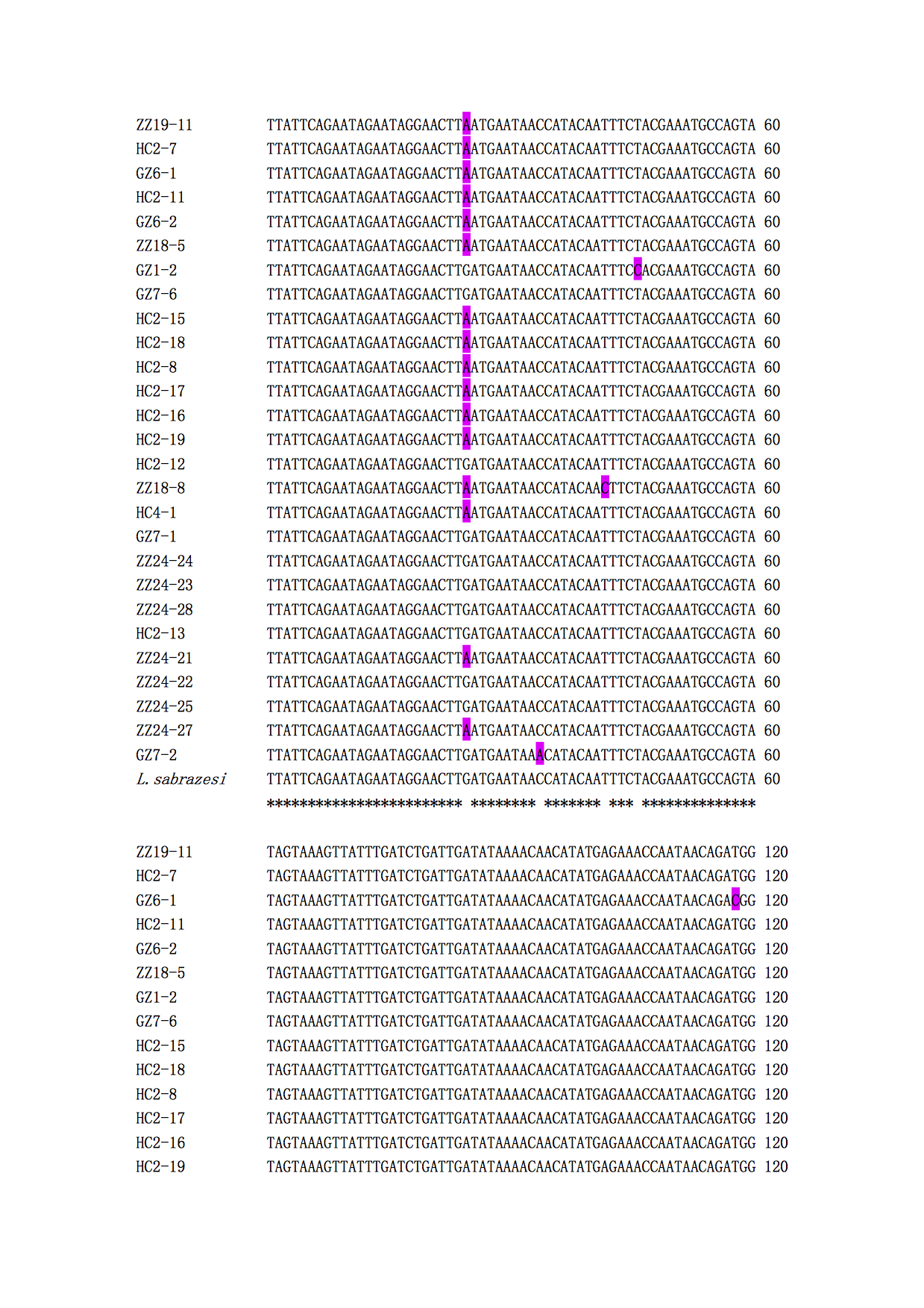

Supplement: Figure S1 — DNA sequence alignments of the Leucocytozoon sabrazesi coxIII gene from chickens obtained from three locations in southern China. (TIFF) [file pone.0094877.s001.tif]

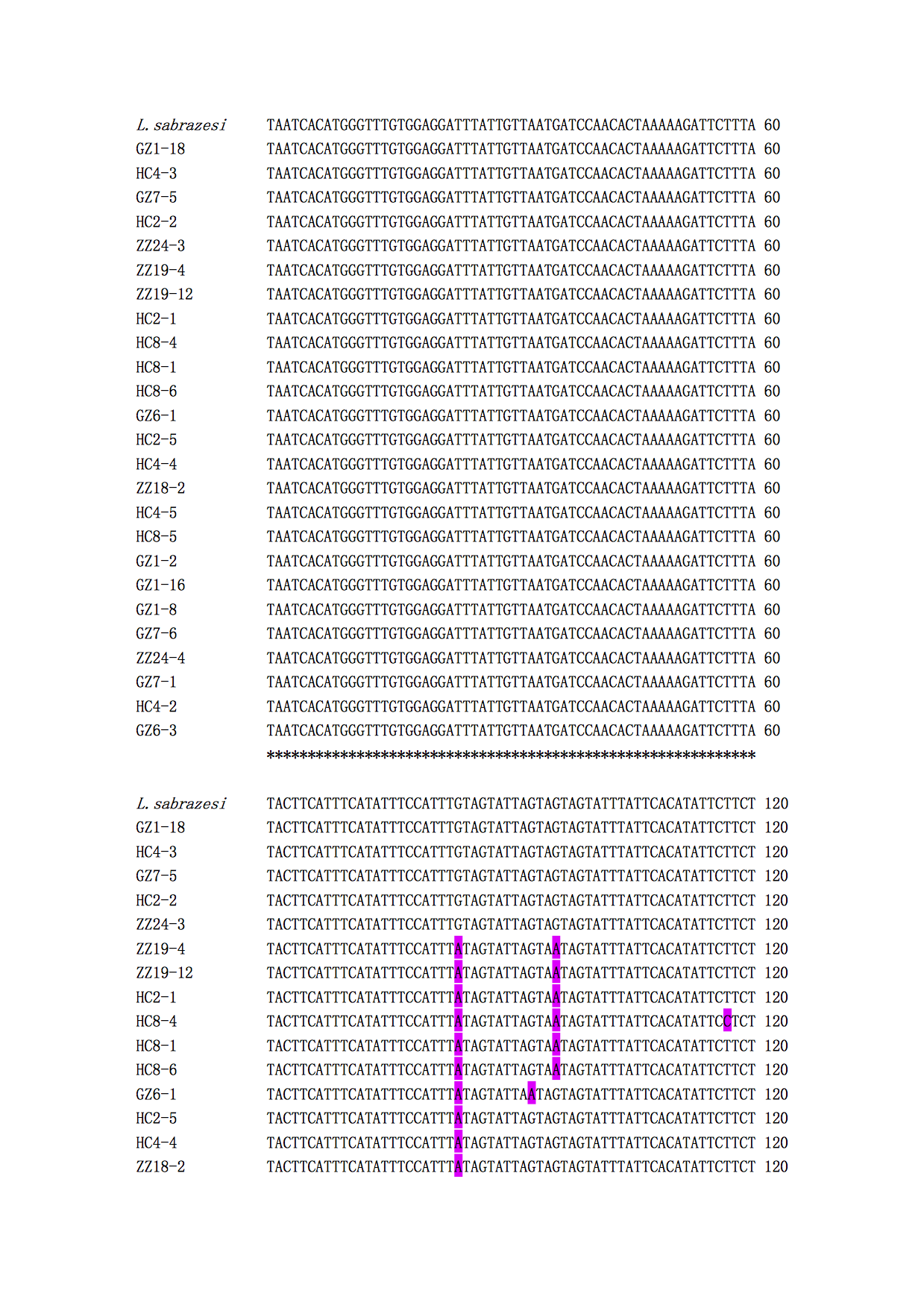

Supplement: Figure S2 — DNA sequence alignments of the Leucocytozoon sabrazesi cytb gene from chickens obtained from three locations in southern China. (TIFF) [file pone.0094877.s002.tif]

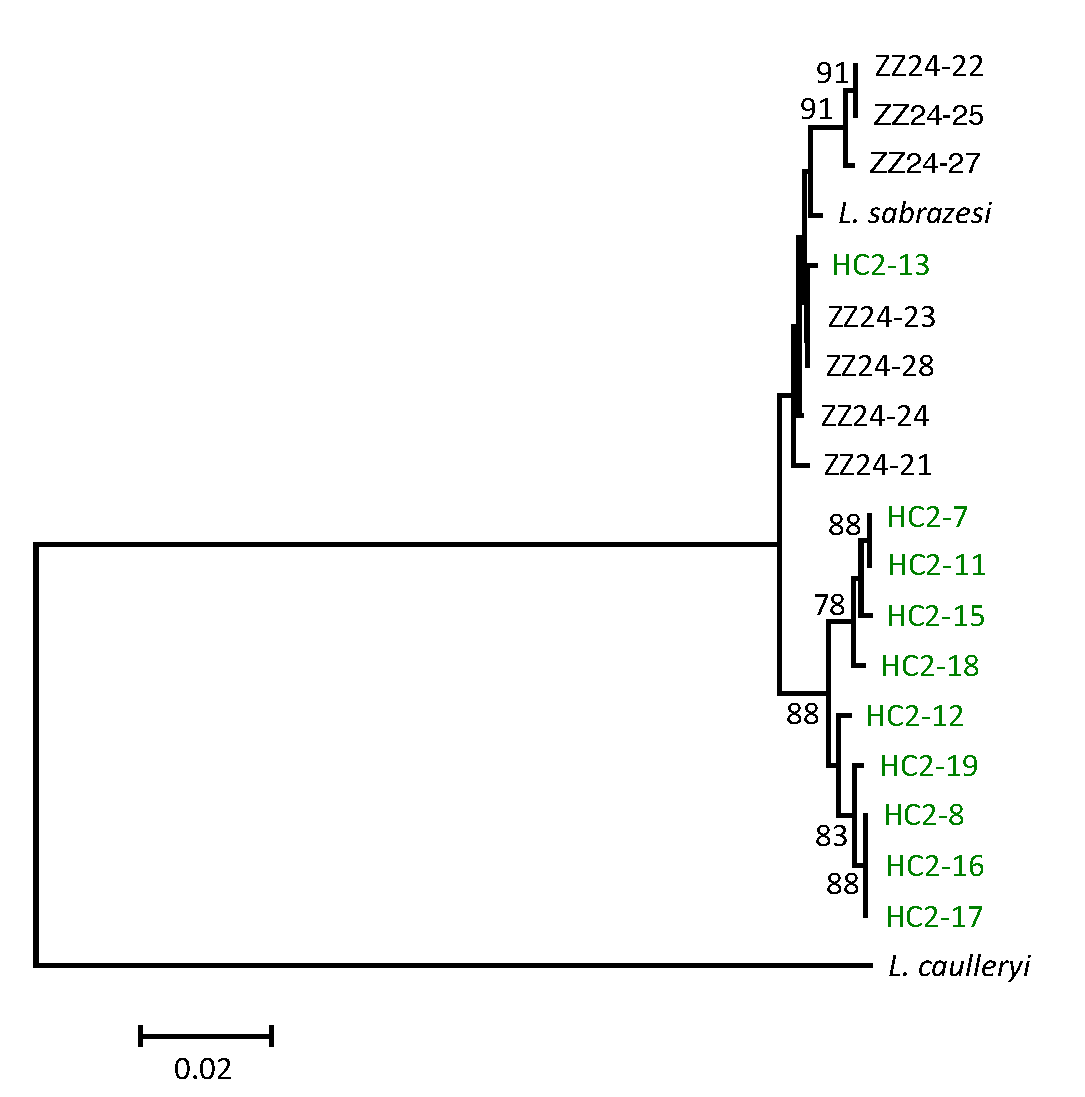

Supplement: Figure S3 — Dendrograms of the coxIII gene segment clustering parasite strains cloned from chickens ZZ24 and HC2. The clustering was performed as in Figure 4 except using the 35 polymorphic sites verified by direct PCR product sequencing. (TIFF) [file pone.0094877.s003.tif]
